# Spectroscopic studies on photodegradation of atorvastatin calcium

**DOI:** 10.1038/s41598-021-94693-5

**Published:** 2021-07-28

**Authors:** Madalina Oprica, Miruna Iota, Monica Daescu, Szilard N. Fejer, Catalin Negrila, Mihaela Baibarac

**Affiliations:** 1 Laboratory of Optical Processes in Nanostructured Materials, National Institute of Materials Physics, Atomistilor Street 405A, P.O. Box MG-7, 77125 Magurele, Romania; 2 Pro-Vitam Ltd., Muncitorilor Street 16, Sfantu Gheorghe, Romania; 3 Nanoscale Condensed Matter Laboratory, National Institute of Materials Physics, Atomistilor Street 405A, P.O. Box MG-7, 77125 Magurele, Romania

**Keywords:** Materials science, Physics

## Abstract

In this work, the photodegradation process of atorvastatin calcium (ATC) is reported as depending on: (1) the presence and the absence of excipients in the solid state; (2) the chemical interaction of ATC with phosphate buffer (PB) having pH equal to 7 and 8; and (3) hydrolysis reaction of ATC in the presence of aqueous solution of NaOH. The novelty of this work consists in the monitoring of the ATC photodegradation by photoluminescence (PL). The exposure of ATC in solid state to UV light induces the photo-oxygenation reactions in the presence of water vapors and oxygen from air. According to the X-ray photoelectron spectroscopic studies, we demonstrate that the photo-oxygenation reaction leads to photodegradation compounds having a high share of C=O bonds compared to ATC before exposure to UV light. Both in the presence of PB and NaOH, the photodegradation process of ATC is highlighted by a significant decrease in the intensity of the PL and photoluminescence excitation (PLE) spectra. According to PLE spectra, the exposure of ATC in the presence of NaOH to UV light leads to the appearance of a new band in the spectral range 340–370 nm, this belonging to the photodegradation products. Arguments concerning the chemical compounds, that resulted in this last case, are shown by Raman scattering and FTIR spectroscopy.

## Introduction

Atorvastatin calcium (ATC) is the active compound from the drugs marketed under the name of Atorvastatin, Torvacard, Sortis, and Atoris. These drugs are administrated in: (1) the cardiovascular disease, such as acute coronary syndromes^1^, atherosclerotic strokes^2^, myocardial infarction^3^; (2) kidney diseases^4^ and (3) diminution of total cholesterol levels^5^. Much effort has been made for detection of ATC in pharmaceutical products and/or biological samples by high-performance liquid chromatography (HPLC)^6^, electrophoresis^7^, square wave voltammetry^8^, photoluminescence (PL)^9^, Raman scattering^10^ UV–VIS spectroscopy^11^ and liquid chromatography-mass spectrometry (LC–MS)^12^.

A topic that has attracted a lot of attention lately in the field of drugs is their photodegradation, when toxic compounds can be formed, and the therapeutic activity of these pharmaceutical compounds can be much diminished^13^. Various photodegradation pathways of ATC^14^ and development of various methods for the isolation of degradation products of ATC^15^ were reported. Until now, the method used for the study of the degradation products of atorvastatin was HPLC^16,17^, LC–MS^18^ and nuclear magnetic resonance (NMR)^19^. According to early studies, the exposure of atorvastatin in water to sunlight induces a photo-oxygenation, which involves the appearance of lactam rings and a shift of the alkyl or aryl functional groups, that were isolated by chromatography^19,20^. In comparison with this study, this work will demonstrated that such a photo-oxygenation process can also take place by exposure of ATC in solid state at the water vapors and oxygen from air. In order to highlight the photodegradation process of ATC in solid state, the studies of photoluminescence (PL), UV–VIS spectroscopy and X-ray photoelectron spectroscopy (XPS) are used.

Generally speaking, for the drugs photodegradation monitoring, the optical methods often used were UV–VIS spectroscopy, XPS spectroscopy and photoluminescence (PL)^21–24^. Recently, using these three methods it has been demonstrated that: (1) azathioprine photodegrades in the presence of oxygen from air^21^; (2) the photodegradation process of folic acid in phosphate buffer (PB) with pH equal to 5.4 is enhanced^22^; and (3) melatonin and acetaminophen, respectively, also are susceptible for photodegradation in the presence of alkaline medium^23,24^.

In the last years, a special attention was given the thermal and oxidative degradation as well as the forced degradation in the presence of the strong acids and alkali media of ATC, the processes studied by LC-MC and HPLC^25,26^. In this work, new optical evidences regarding the photodegradation process of ATC, in the presence of alkaline medium as well as phosphate buffer (PB) with pH 7 and 8, respectively, will be reported by PL, UV–Vis spectroscopy, Raman scattering and FTIR spectroscopy. The influence of the excipients on the ATC photodegradation will be shown, too. Chemical mechanism proposed during ATC photodegradation will be explained taking into account the changes induced in the XPS, Raman and FTIR spectra.

## Results and discussion

### Photoluminescence properties of ATC

**Figure 1:**
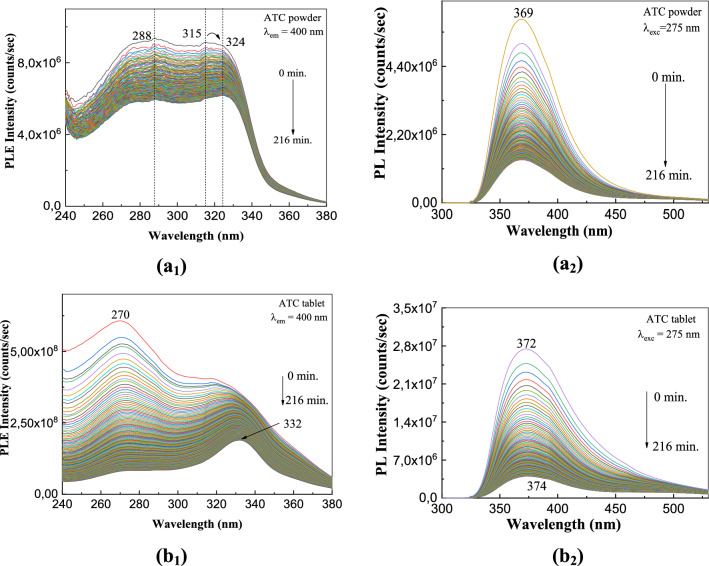
PLE (1) and PL (2) spectra of ATC in powder state (**a**_**1**_, **a**_**2**_) and as tablet (**b**_**1**_, **b**_**2**_) before and after exposure to UV light for 216 min.

The photodegradation process of ATC is one that can occur both in the preparation of tablets and in their handling by patients who have been administered drugs with such compounds in the therapeutic regimen. A method that allows monitoring the process of photodegradation of ATC both in the solid and liquid state is PL and PLE as shown in the following. In this context, Fig. 1 shows the PLE and PL spectra of ATC in the powder and tablet state, in the initial state and after exposure to UV light for 216 min.

According to Fig. 1a_1_, the PLE spectrum of ATC in powder state is characterized by two bands having maxima at 288 nm and 315 nm, whole the ratio between their intensities is equal to 1.03. The PL spectrum of ATC in powder state shows an emission band with maximum at 369 nm (Fig. 1a_2_). In contrast with the ATC in powder state, for the ATC tablet one observes that: (1) the PLE spectrum shows two bands at 270 nm and 318 nm, the ratio between their intensities being equal to 1.5 (Fig. 1b_1_) and (2) the PL spectrum shows a band, with the maximum at 372 nm, which was asymmetric towards smaller energies (Fig. 1b_2_). As the exposure time to UV light is increased, the following changes are remarked in Fig. 1: (1) a shift of the band at 315–324 nm in Fig. 1a_1_, accompanied of a change of the ratio between their intensities from 1.03 to 0.96; (2) the intensity of the emission band at 369 nm of ATC in powder state decreases from 5.9 × 10^6^ to 1.39 × 10^6^ counts/s (Fig. 1a_2_); (3) a shift of the band of the PLE spectrum of ATC tablet from 318 to 332 nm, simultaneous with the change of the ratio between the intensities of the two bands at 270 nm and 318–332 nm from 1.5 to 0.43 (Fig. 1b_1_); and (4) a progressive decrease in the intensity of the emission band at 372 nm from 2.74 × 10^7^ to 4.02 × 10^6^ counts/s (Fig. 1b_2_). According to these results, regardless if ATC was in a powder or tablet formulation, a photodegradation process occurs.

According to Fig. 1S, the photodegradation process of the ATC powder also occurs in the presence of excipients. In this context, we note that: (1) the PLE spectrum of TOR (Fig. 1Sa_1_) shows two bands with maxima at 227 nm and 319 nm, whose the intensities vary from 1.14 × 10^8^ and 1.4 × 10^8^ counts/s to 2.73 × 10^7^ and 4.7 × 10^7^ counts/s, respectively, before and after the exposure of the sample to UV light for 216 min; a similar behavior is noted in the case of the PLE spectrum of SOR (Fig. 1Sb_1_), which shows two components having the maxima at 319 nm and 282 nm whose intensities is changed from 1.25 × 10^8^ and 1.17 × 10^8^ counts/s to 6.1 × 10^8^ and 4.11 × 10^7^ counts/s, respectively, before and after the exposure of the sample to UV light for 216 min; and (2) the PL spectra of TOR (Fig. 1Sa_2_) and SOR (Fig. 1Sb_2_) are characterized by an emission band having maximum at 369 nm and 370 nm. Before the exposure to UV light of TOR and SOR, the intensities of PL bands are equal to 1.82 × 10^6^ and 1.33 × 10^5^ counts/s. After the exposure of TOR and SOR to UV light, for 216 min, the intensity of the PL bands becomes equal to 3.35 × 10^5^ and 7.62 × 10^4^ counts/s. The lower values of the intensity of the PL bands of TOR (Fig. 1Sa_2_) and SOR (Fig. 1Sb_2_) compared to ATC (Fig. 1a_2_), both before and after exposure to UV light for 216 min, demonstrate that the presence of excipients induces an ATC PL quenching process. In our opinion, the variations in the intensities of the PLE and PL spectra of the ATC, TOR and SOR origin both in the interaction of ATC with the water vapor and oxygen from air, when ATC is transformed into photo-oxygenation compounds shown in Fig. 3S, and the generation of a charge-transfer complex between benzene rings and oxygen as reported by Morikawa et al.^27^ and Gooding et al.^28^.

**Figure 2:**
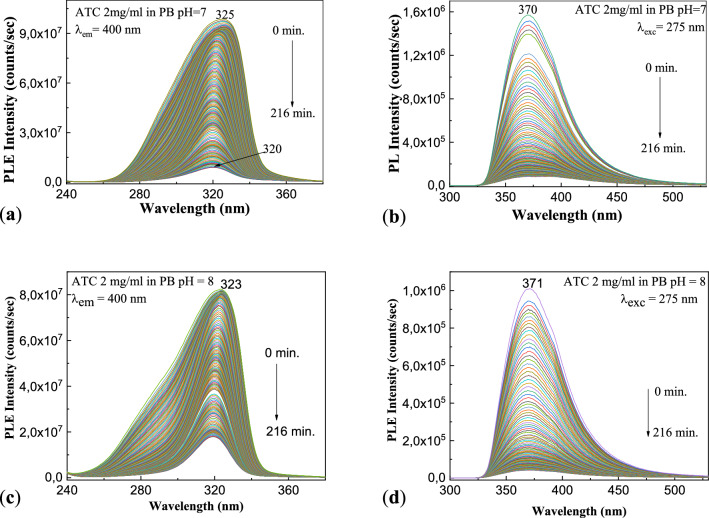
PLE and PL spectra ale ATC in initial state and after exposure at UV light, in the presence of PB with pH equal to 7 (**a**, **b**) and 8 (**c**, **d**), when the emission wavelength is equal to 400 nm and the excitation wavelength is equal to 275 nm.

In the order to support this hypothesis, Fig. 2S illustrates the PLE and PL spectra of the aqueous solution of ATC and the dependence of the photodegradation process with the concentration of ATC. As the concentration of ATC in aqueous solution increases from 1 to 2 mg/ml, one observes that: (1) in the initial state, the intensities of the PLE and PL spectra decrease from 1.36 × 10^8^ counts/s (Fig. 2Sa_1_) and 1.7 × 10^8^ counts/s (Fig. 2Sa_2_) to 1.18 × 10^8^ counts/s (Fig. 2Sb_1_) and 1.18 × 10^8^ counts/s (Fig. 2Sb_2_), respectively; and (2) as increasing the exposure time to UV light up to 216 min, the intensities of the PLE and PL spectra decrease at 8.14 × 10^7^ counts/s (Fig. 2Sa_1_) and 8.67 × 10^5^ counts/s (Fig. 2Sa_2_) to 7.25 × 10^7^ counts/s (Fig. 2Sb_1_) and 5.91 × 10^4^ counts/s (Fig. 2Sb_2_), respectively. Taking into account the above values of the PLE and PL spectra intensities, before (I^0^_PLE_ and I^0^_PL_) and after exposure to UV light (I_PLE_ and I_PL_), one can conclude that in the case of the aqueous solution of ATC having the concentration 1 mg/ml and 2 mg/ml: (1) the I^0^_PLE_/I_PLE_ ratio is equal to 1.67 (Fig. 2Sa_1_) and 1.63 (Fig. 2Sb_1_); and (2) the I^0^_PL_/I_PL_ ratio is equal to 1.96 (Fig. 2Sa_2_) and 20.82 (Fig. 2Sb_2_). In this last case, i.e., for the aqueous solution of ATC with the concentration of 2 mg/ml, under UV light, a shift of the emission band from 376 to 389 nm is also reported.

**Figure 3:**
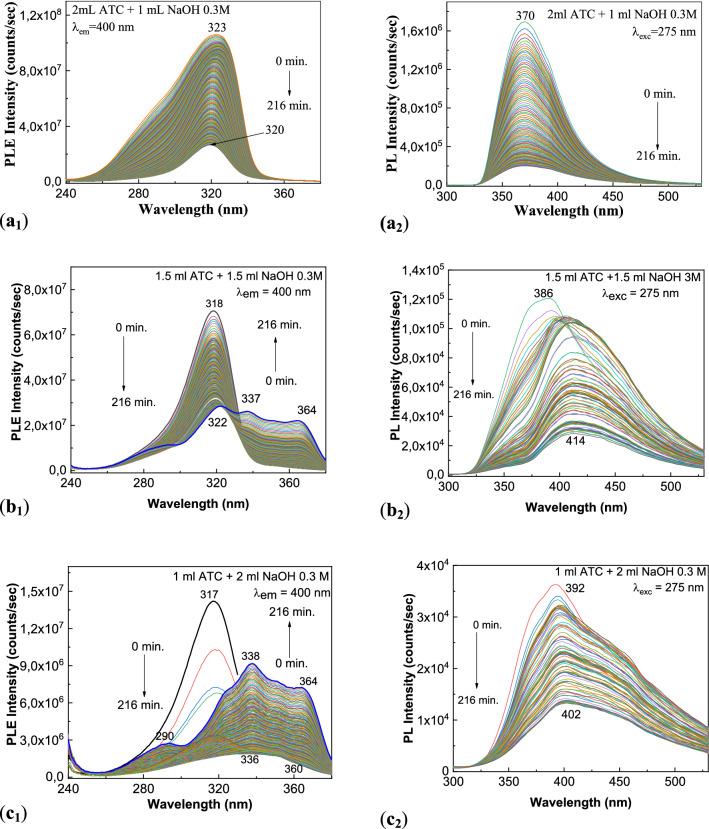
PLE (1) and PL (2) spectra of the aqueous solutions of: 2 ml ATC 2 mg/ml interacted with 1 ml NaOH 0.3 M (**a**_**1**_, **a**_**2**_); 1.5 ml ATC 2 mg/ml interacted with 1.5 ml NaOH 0.3 M (**b**_**1**_, **b**_**2**_); and 1 ml ATC 2 mg/ml interacted with 2 ml NaOH 0.3 M (**c**_**1**_, **c**_**2**_) before and after the exposure at UV light for 216 min.

**Figure 4:**
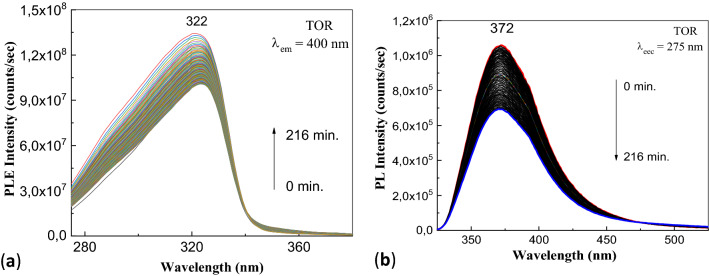
PLE (**a**) and PL (**b**) spectra of the aqueous solutions of ATC 2 mg/ml in the presence of excipients form the drug marked under the name TOR and the influence of the UV light by exposure for 216 min.

Figure 3S shows the mechanism of the photodegradation reaction of ATC in the presence of the water vapors and oxygen, when ATC is exposed to UV light. This mechanism involves: (1) in the first stage, the reaction of atorvastatin calcium with water vapors, when result atorvastatin and Ca(OH)_2_ and (2) in the second stage, the interaction of atorvastatin with oxygen when the photo-oxygenation reaction leads to the generation of four compounds such as those shown in reaction 2 of Fig. 3S. According to Fig. 3S, by the exposure of ATC to UV light in the presence of the oxygen and water vapors from air leads to an increase of the C=O groups weight in the photodegradation products. In order to sustain this sentence, in Fig. 4S are shown the XPS C1s, O1S, N1S and Ca2p spectra of ATC before and after the exposure to UV light. According to Fig. 4Sa_1_, the deconvolution of the XPS C1s spectrum of ATC in the initial state highlights six peaks at 283.9, 285, 285.6, 286.3, and 287.8 eV assigned to the C–C, C=C and C–H bonds in aromatic ring, the C–N bond in aromatic ring, the C–N bond in the –NH–CO– amide functional group, the C–OH bond, and sp^2^ C–F bond, respectively^29^. The peak at 290.7 eV of low intensity corresponds to a shake-unspecific transition π–π*^29^. Figure 4a_2_ highlights that after the exposure of ATC to UV light, the appearance of a new peak of low intensity at 292.1 eV is assigned to π–π* shake-up sattellite of sp^2^ C atom^29^. The exposure to UV light of ATC sample is observed that not induces major changes in the XPS N1s, Ca2p and F1s spectra (Fig. 4Sc_1_–Se_2_). Not the same thing happens with the XPS O1s spectrum, when ATC is exposed to UV light (Fig. 4Sb_1_,Sb_2_). By the deconvolution of the XPS O1s spectrum of ATC, before the exposure to UV light, highlights two peaks at 530.9 and 532.3 eV, assigned to the C=O and C–OH bonds^29^, respectively (Fig. 4Sb_1_). The careful analysis of the Fig. 4Sb_1_,Sb_2_ shows a change of the ratio between the intensities of the peaks at 530.9 and 532.3 eV from 1.4 to 2.1. This variation pleads for the generation of new C=O bonds, the result which confirms reactions shown in Fig. 3S.

### Photodegradation of ATC in the presence of PB

ATC-induced liver dysfunction has induced the development of new sensory platforms for its detection in pharmaceutical and urine samples. In this order, ATC was dissolved in PB with a pH range of 3.0–11.2^30^. An essential parameter in such applications is the knowledge of the behavior of these solutions in the presence of UV light. In the following, the influence of UV light on ATC solutions in PB with pH equal to 7 and 8 will be monitored by PL. Figure 2 shows the dependence of the PL spectra of the ATC solution having the concentration of 2 mg/ml with the pH of PB. Regardless the pH value of the solution of ATC in PB, before the exposure to UV light, the maxima of the PLE and PL spectra are localized at 323–325 nm and 370–371 nm, respectively.

Depending on the pH of PB, i.e. 7 and 8, in Fig. 2 one observes that: (1) before of the exposure to UV light of the ATC solutions, the intensities of PLE spectra are equal to 9.86 × 10^7^ counts/s (Fig. 2a) and 8.24 × 10^7^ counts/s (Fig. 2c), when the ATC is dissolved in PB with the pH equal to 7 and 8, respectively; the intensity of PL spectra of the solutions of ATC 2 mg/ml in PB with pH equal to 7 and 8, respectively, varies from 1.57 × 10^6^ counts/s (Fig. 2b) to 1.01 × 10^7^ counts/s (Fig. 2d); (2) after exposure to UV light for 216 min, one observes a significant decrease of the intensities of the PLE spectra up to 9.03 × 10^6^ counts/s (Fig. 2a) and 1.8 × 10^7^ counts/s (Fig. 2c), accompanied of an up-shift of PLE bands from 325/323 to 320/319 nm (Fig. 2a/c). A similar behavior is noted also in the case of the PL spectra. Thus, in the case of ATC in PB with pH equal to 8, a decrease in the intensity of the emission band from 1.01 × 10^7^ to 4.25 × 10^4^ counts/s, accompanied of a shift of the maximum of PL band from 323 to 319 nm take place (Fig. 2d). In the case of ATC in PB with pH equal to 7 one remarks that the intensity of PL band from 370 nm decreases from 1.57 × 10^6^ to 8.55 × 10^4^ counts/s, simultaneously with the appearance of a new band with the maximum at 392 nm having the intensity equal to 8.6 × 10^4^ counts/s (Fig. 2b). Such a behavior of PL spectra was also reported in the case of the acetate buffer^31^. The variations reported in Fig. 2 indicate an intensification of the photodegradation process of ATC in the presence of PB with pH equal to 7 and 8, respectively, compared to those shown in Fig. 2Sb_1_,Sb_2_. In order to explain this result, we consider: (1) reaction 1 of Fig. 3S which illustrates that the interaction of ATC with H_2_O leads to atorvastatin and Ca(OH)_2_; and (2) the interaction of Ca(OH)_2_ with PB which takes place according to the following two reactions, these involving the generation of the compounds CaHPO_4_, Ca(HPO_4_)_2_ and NaOH.

$$\begin{aligned} & {\text{Ca}}\left( {{\text{OH}}} \right)_{2} + {\text{Na}}_{2} {\text{HPO}}_{4} \to {\text{CaHPO}}_{4} + 2{\text{NaOH}} \\ & {\text{Ca}}\left( {{\text{OH}}} \right)_{2} + 2{\text{NaH}}_{2} {\text{PO}}_{4} \to {\text{Ca}}\left( {{\text{H}}_{2} {\text{PO}}_{4} } \right)_{2} + 2{\text{NaOH}} \\ \end{aligned}$$

As will be highlighted in the next section of this paper, further photodegradation of ATC is induced in the presence of the aqueous NaOH solution.

### Photodegradation of ATC in the presence of alkaline solution

**Figure 5:**
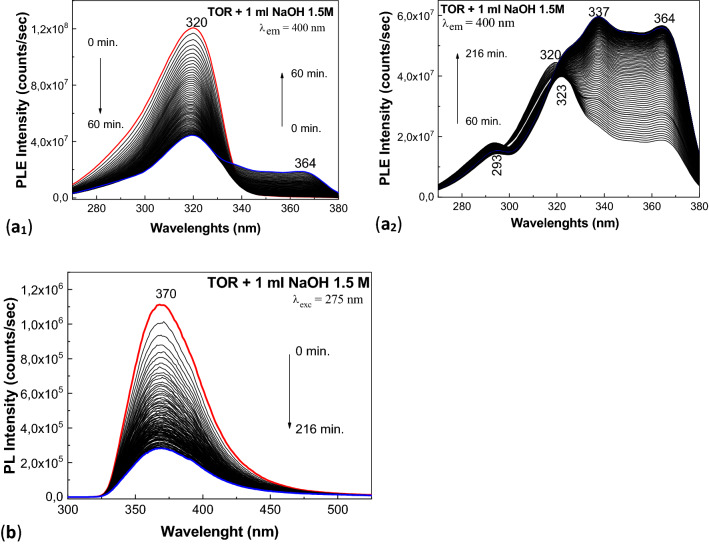
PLE (**a**_**1**_, **a**_**2**_) and PL(**b**) spectra of the aqueous solutions of 2 ml ATC 2 mg/ml in the presence of excipients form the drug marked under the name TOR interacted with 1 ml NaOH 1.5 M and the influence of the UV light by exposure, time of 216 min.

The alkaline stress induces to atorvastatin was studied by LC–MS beginning with 2008^25^. New results obtained from correlated studies of PL, Raman scattering and IR spectroscopy on ATC photodegradation in the presence of NaOH are presented below. Thus, Figs. 3 and 5S highlight the interaction of ATC with the solutions of NaOH 0.3 M and 1.5 M, respectively. As increasing of the weight of alkaline agent in the mass of the two reactants, ATC and NaOH, in the initial state one observes in Fig. 3a_1_–c_1_ a gradual decrease in the intensity of the PLE spectra from 1.06 × 10^8^ to 7 × 10^7^ counts/s and 3.36 × 10^6^ counts/s, respectively. A similar behavior occurs in the case of PL spectra, the diminution in the intensity of the emission band at 370 nm taking place from 1.16 × 10^6^ to 1.2 × 10^5^ counts/s and 3.67 × 10^4^ counts/s, respectively (Fig. 3a_2_–c_2_).

As increasing the exposure time to UV light up to 216 min and of the weight of alkaline agent in the mass of the two reactants, ATC and NaOH, Fig. 3 reveals a decrease in the intensity of PLE spectra at 2.66 × 10^7^ counts/s (Fig. 3Sa_1_), 2.84 × 10^7^ counts/s (Fig. 3Sb_1_) and 9.16 × 10^6^ counts/s (Fig. 3Sc_1_) as well as of the intensity of the PL spectra at 2.01 × 10^5^ counts/s (Fig. 3Sa_2_), 2.82 × 10^4^ counts/s (Fig. 3Sb_2_) and 1.41 × 10^4^ counts/s (Fig. 3Sc_2_). A careful analysis of: (1) the PLE spectra highlights that as increasing the NaOH weight added to the ATC solution, under UV light, in Fig. 3Sb_1_,Sb_2_ appear new bands with the maximum at 337 nm and 364 nm, while in the case of (2) the PL spectra one remarks that after 216 min of exposure to UV light, a down-shift of the maximum of the emission bands from 386 nm (Fig. 3Sb_2_) and 392 nm (Fig. 3Sc_2_) to 414 nm (Fig. 3Sb_2_) and.402 nm (Fig. 3Sc_2_) takes place.

The increase of the concentration of NaOH at 1.5 M induces changes similar with those reported in Fig. 5S. Thus, in Fig. 5S one observes that in the case of the ATC interacted with NaOH 1.5 M, by the exposure to UV light, time of 216 min, takes place: (1) a variation in the intensity of the PLE band at 320–317 nm from 9.71 × 10^7^ to 1.94 × 107 counts/s (Fig. 5Sa); and (2) a down-shift of the PL band from 380 to 393 nm and a decrease of the intensity from 1.29 × 10^5^ to 1.86 × 10^4^ counts/s (Fig. 5Sb). Such variations are also observed in the case of pharmaceutical products of the type TOR and SOR.

Before reveal the influence of the alkaline medium, the photodegradation process of the aqueous TOR solution is shown. Figure 4 shows the PLE and PL spectra of the aqueous solution of TOR.

According to Fig. 4a, the PLE spectra show a maximum at 324 nm, whose intensity decreases from 1.34 × 10^8^ to 1.01 × 10^8^ counts/s, as increasing the exposure time to UV light time of 216 min The PL spectrum of the aqueous solution of TOR is characterized by a band with the maximum at 372 nm, whose intensity decreases from 1.06 × 10^6^ to 6.92 × 10^5^ counts/s, as increasing the exposure time to UV light up to 216 min (Fig. 4b).

The changes induced during the interaction of TOR with NaOH, under UV light, are shown in Fig. 5.

Figure 5a_1_,a_2_ shows the PLE spectra of 2 ml TOR 2 mg/ml interacted with 1 ml NaOH 1.5 M, when the aqueous solution is exposed to UV light for 216 min. In the initial state, the PLE spectrum shows a band with a maximum at 320 nm having the intensity equal to 1.21 × 10^8^ counts/s. As the exposure time to UV light increases to 60 min, Fig. 5a_1_ illustrates a decrease of the intensity of the band at 320 nm up to 4.44 × 10^7^ counts/s simultaneous with the appearance of other two bands having maxima at 293 nm and 364 nm, whose intensity is equal to 1.4 × 10^7^ counts/s and 1.88 × 10^7^ counts/s, respectively. According to Fig. 5a_2_, the increase of the exposure time to UV light up to 216 min induces: (1) a down-shift of the band from 320 to 323 nm and an increase in the intensity up to 4.65 × 10^7^ counts/s; (2) the appearance of the band at 337 nm, and its increase in the intensity, so that in the final state the intensity becomes 5.97 × 10^7^ counts/s; and (3) an additional increase in the intensity of the band at 364 nm up to 5.66 × 10^7^ counts/s. Figure 5b shows PL spectrum of 2 ml TOR 2 mg/ml interacted with 1 ml NaOH 1.5 M, when the aqueous solution is irradiated with UV light for 216 min. Similar to ATC, the PL spectrum of TOR shows an emission band at 370 nm, whose intensity decrease from 1.1 × 10^6^ to 2.83 × 10^5^ counts/s, when the exposure time to UV light increases up to 216 min.

**Figure 6:**
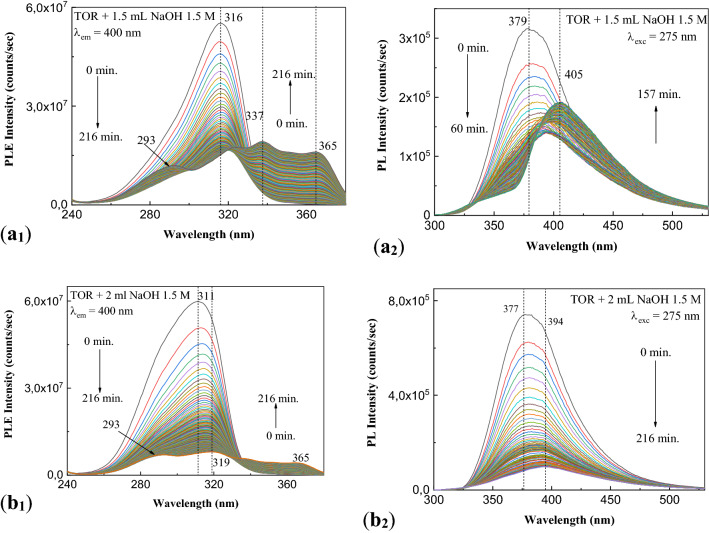
PLE (1) and PL (2) spectra of the aqueous solutions of: (1) 1.5 ml TOR interacted with 1.5 ml NaOH 1.5 M (**a**_**1**_, **a**_**2**_) and (2) 1 ml TOR interacted with 2 ml NaOH 1.5 M (**b**_**1**_, **b**_**2**_), before and after the exposure to UV light, time of 216 min.

In Fig. 6 one observes that as increasing the NaOH weight in the presence of aqueous solution of TOR, a similar behavior with that reported in Fig. 5 is remarked under UV light. In this case, (1) Fig. 6a_1_ indicates that in the initial state the solution of TOR interacted with NaOH shows a maximum at 316 nm, whose intensity is equal to 5.52 × 10^7^ counts/s; as increasing the exposure time at UV light up to 216 min, the PL spectrum contains four bands with the maxima at 292 nm, 320 nm, 337 nm and 365 nm, whose intensities become equal to 1.2 × 10^7^ counts/s, 1.66 × 10^7^ counts/s, 1.93 × 10^7^ counts/s and 1.61 × 10^7^ counts/s, respectively; (2) Fig. 6a_2_ shows an emission band at 379 nm, whose intensity is equal to 3.16 × 10^5^ counts/s, and which under UV light in the first 60 min leads to a down-shift at 394 nm, the intensity of PL band becoming equal to 1.39 × 10^5^ counts/s; the increase of the exposure time at UV light induces an additional down-shift of PL band at 407 nm so that after 216 min of exposure to UV light the intensity is equal to 1.92 × 10^5^ counts/s; (3) Fig. 6b_1_ highlights a band with the maximum at 311 nm, whose intensity is equal to 5.98 × 10^7^ counts/s; according to Fig. 6b_1_, after 216 min of exposure to UV light, four bands with the maxima at 293 nm, 319 nm, 337 nm and 365 nm, having intensities equal to 7.03 × 10^6^ counts/s, 8.06 × 10^6^ counts/s, 5.77 × 10^6^ counts/s and 4.29 × 10^6^ counts/s, respectively, are observed; and (4) Fig. 6b_2_  evidences an emission band with the maximum at 377 nm having the intensity equal to 7.41 × 10^5^ counts/s, which under UV light is down-shifted to 393 nm, when its intensity becomes equal to 9.831 × 10^4^ counts/s.

**Figure 7:**
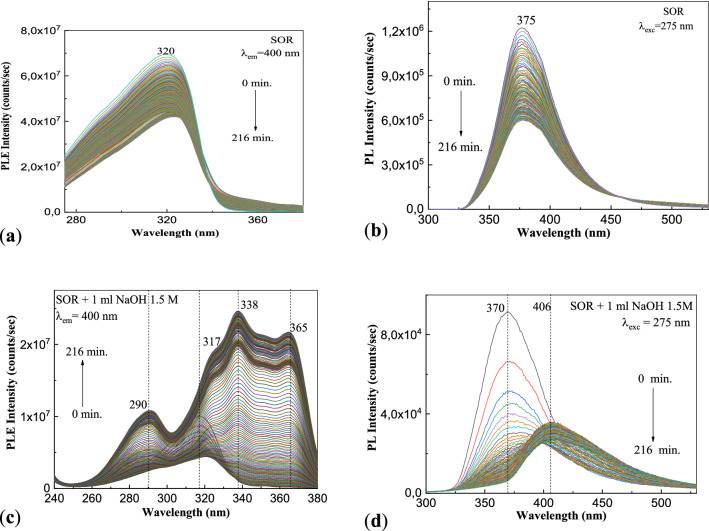
PLE and PL spectra of the aqueous solutions of 3 ml SOR (**a**, **b**) and 2 ml SOR interacted with 1 ml NaOH 1.5 M (**c**, **d**) before and after the exposure to UV light for 216 min.

Figure 7 shows the PLE and PL spectra of SOR before and after the interaction with NaOH and the influence of the UV light.

According to our expectation, in the initial state, i.e. before to exposure to UV light: (1) the PLE spectrum of SOR shows a band with the maximum at 320 nm having the intensity equal to 6.95 × 10^5^ counts/s (Fig. 7a), while the PL spectrum shows an emission band with the maximum at 377 nm having the intensity equal to 1.22 × 10^6^ counts/s (Fig. 7b), and (2) the PLE and PL spectra of SOR interacted with NaOH show bands with the maxima at 317 nm and 370 nm, having intensities equal to 9.95 × 10^6^ counts/s (Fig. 7c) and 9.16 × 10^4^ counts/s (Fig. 7d). The exposure to UV light of the aqueous solution of SOR before and after interaction with NaOH leads to the following variations in: (1) Fig. 7a, a down-shift of the PLE band from 320 to 323 nm and a decrease in the intensity up to 4.2 × 10^7^ counts/s; (2) Fig. 7b, a decrease in the intensity of the emission band at 377 nm up to 6.01 × 10^6^ counts/s; (3) Fig. 7c, a down-shift of the PLE band from 317 to 324 nm simultaneous with the apparition of new bands at 290 nm, 338 nm and 365 nm, their intensities being equal to 1.86 × 10^7^ counts/s, 1.08 × 10^7^ counts/s, 2.46 × 10^7^ counts/s, and 2.17 × 10^7^ counts/s, respectively; and (4) Fig. 7d, a gradual down-shift of the emission band from 370 to 399 nm and 407 nm, when a decrease in the intensity of the PL band from 9.16 × 10^4^ to 2.55 × 10^4^ counts/s and 3.47 × 10^4^ counts/s, respectively, is reported. Summarizing the results presented in Figs. 6 and 7, they indicate that the photodegradation process of ATC in the presence of NaOH is not inhibited by the presence of excipients.

**Figure 8:**
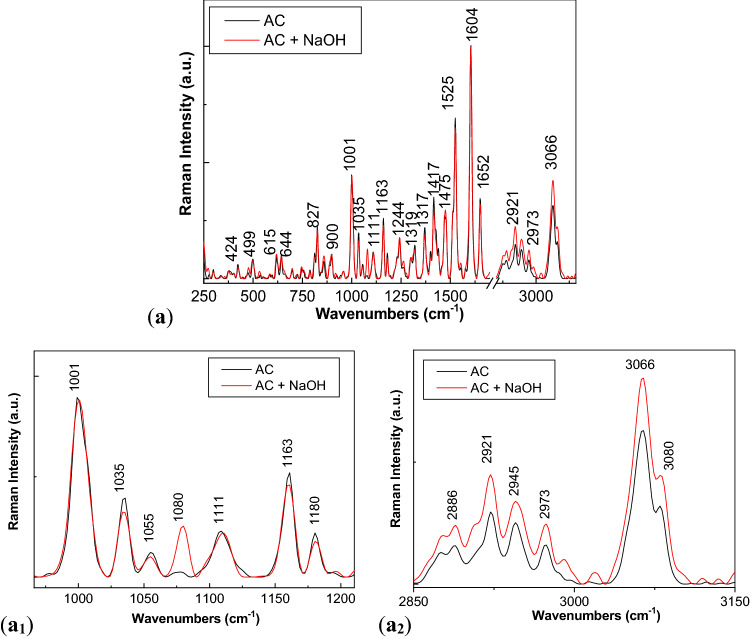
Raman spectra of ATC interacted with NaOH (**a**). (**a**_**1**_, **a**_**2**_) show spectral ranges 950–1250/cm and 2850–3150 cm^−1^.

**Figure 9:**
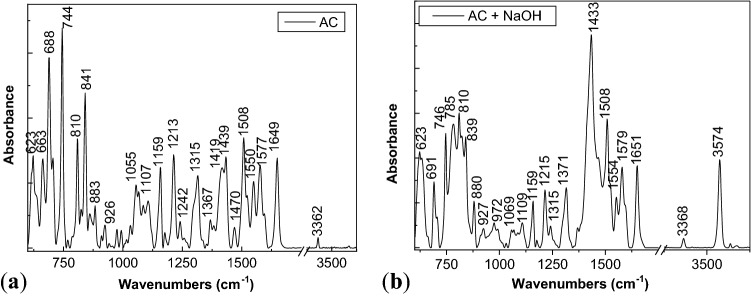
IR spectra of ATC before (**a**) and after interaction with NaOH (**b**).

The mechanism of the photodegradation reaction of ATC in the presence of NaOH is shown in Fig. 6S. This involves: (1) in the first stage, the formation of atorvastatin sodium and Ca(OH)_2_; and (2) in the second stage, the hydrolysis reaction will take place the breaking of the amide group, when an amine group and a carboxyl group will results. In order to prove that Fig. 6S explains changes reported in Figs. 3, 4, 5, 6, 7 and 5S, in the following the IR and Raman spectra of ATC before and after the interaction with NaOH are shown in Figs. 8 and 9.

Figure 8a shows the main Raman lines of ATC, which are situated at 424, 499–615, 644, 827, 900, 1001–1035–1111, 1163, 1244, 1319, 1371, 1417, 147–1525, 1604, 1652, 2886–2921–2945–2973 and 3066–3080 cm^−1^, these being assigned to the vibrational modes: in-plane OCO rocking + out-of-plane benzene deformation, C–C–C deformation, O–H out of plane deformation, C–H out of plane deformation, CH wagging in benzene ring + C–N–C stretching in amide group + C–C–O stretching, C–C–H in plane deformation, C–C–H in plane deformation + O–H in plane deformation, C–N stretching, CH_3_/CH_2_ deformation symmetrical, CH_3_ deformation, C=C stretching in benzene ring, C–C stretching in benzene ring, C=O stretching, C–H symmetrical stretching in CH_2_ and CH_3_ groups and NH stretching + OH asymmetrical stretching^32–35^. According to Fig. 8a_1_,a_2_, the interaction of AC with NaOH induces in Raman spectra the following changes: (1) the appearance of a new line with the maximum at 1080 cm^−1^ and (2) an increase in the intensities of the lines situated in the spectral range 2850–3150 cm^−1^. An explanation for the increase in the intensity of the Raman line at 3066–3080 cm^−1^, assigned to the vibrational mode of NH stretching + OH asymmetrical stretching, can be given only if we accept that interaction of ATC with NaOH takes place according to reactions 1 and 2 of Fig. 6S, when new N–H bonds and carboxyl groups are resulted.

Complementary data are reported in Fig. 9. According to Fig. 9a, the main IR bands of ATC are peaked at ~ 623, 663, 688–744, 810–841–883–927, 1055, 1107, 1159, 1213, 1316, 1419–1439, 1508, 1550, 1577–1649 and 3362 cm^−1^, they being assigned to the vibrational modes of C–C–C deformation in benzene ring, O–H out of plane deformation, C–H out of plane deformation, C–F stretching, C–C–H in plane deformation, C–C–H in plane deformation + OH in plane deformation, C–N/C–O stretching, CH_3_/CH_2_ symmetrical deformation, CH_3_ deformation, C=C stretching in benzene ring, C–C stretching in benzene ring, C=O stretching in amide group, and N–H stretching^35–39^.

The interaction of ATC with NaOH has induced in IR spectra: (1) the disappearance of the IR band at 663 cm^−1^; (2) a change of the absorbance of the IR bands at 623 cm^−1^ and 688–691 cm^−1^ from 0.48 (Fig. 9a) to 1.52 (Fig. 9b) as well as of the IR bands at 810 cm^−1^ and 839−840 cm^−1^ from 0.7 (Fig. 9a) to 1.2 (Fig. 9b); (3) a down-shift of the IR band from 1439 cm^−1^ (Fig. 9a) to 1433 cm^−1^ (Fig. 9b), simultaneous with its increase in the absorbance; (4) a change of the ratios between the absorbances of the IR bands peaked at: (a) 1508 cm^−1^ and 1577 cm^−1^ (A_1508_/A_1577_) from 1.33 (Fig. 9a) to 1.6 (Fig. 9b); (b) 1508 cm^−1^ and 1649 cm^−1^ (A_1508_/A_1649_) from 1.22 (Fig. 9a) to 1.57 (Fig. 9b); and (c) 880–883 cm^−1^ and 1213–1215 cm^−1^ from 0.47 (Fig. 9a) to 0.8 (Fig. 9b); (5) an increase in the absorbance of the IR band peaked at 1159 cm^−1^ attributed to the C–N/C–O stretching vibrational mode, and (6) the appearance of a new IR band peaked at 3574 cm^−1^ assigned to the carboxyl group stretching^39^ (Fig. 9b). A puzzling fact is that the IR bands at 880 cm^−1^ and 3574 cm^−1^ are often assigned to the out-of-plane NH bending and NH stretching in primary amine^22^. The presence of IR bands at 880 cm^−1^ and 3574 cm^−1^ as well as the increase in the absorbance of the IR band at 1159 cm^−1^ proves that generation of new NH bonds and carboxyl groups as shown Fig. 6S.

## Conclusions

In this work, new results concerning the ATC photodegradation were reported by photoluminescence, Raman scattering and FTIR spectroscopy. Our results allow to conclude that: (1) according to our PLE and PL studies, the photodegradation process of ATC takes place both in the powder and table state, under UV light; in the case of this process, a significant role is played of the water vapors and oxygen from air, which transform ATC into atorvastatin and successively to generation of the four photodegradation products identified previously by HPLC; (2) the presence of the excipients not inhibit the photodegradation process, when ATC is in solid state or as aqueous solution; (3) in the presence of PB with pH 7 and 8, the ATC photodegradation process is highlighted by a gradual decrease in the intensity of the PLE and PL spectra, as a consequence of the generation of atorvastatin and Ca(OH)_2_; the interaction of Ca(OH)_2_ with the constituents of PB induces the generation of NaOH, which will intensified the photodegradation process of atorvastatin; and (4) in the presence of NaOH, the photodegradation process of ATC induces to the appearance of new bands at 293 and 364 nm in PLE spectra, simultaneous with decrease in the intensity of the PL spectra; using FTIR spectroscopy and Raman scattering, we have demonstrate that the ATC hydrolysis reaction leads to the generation of new compounds which contain N–H bonds in primary amine groups as well as carboxylic groups, as shown in Fig. 6S. These results indicate that PL and PLE can be adequate methods in the monitoring and the understanding of the oxidation processes of ATC. This study clearly demonstrates that the preservation of medicines containing ATC must take place in the absence of oxygen and UV light.

## Methods

ATC, Na_2_HPO_4_ and NaH_2_PO_4_ as well as NaOH were bought from Sigma Aldrich. The drugs marketed under the name of Torvacard (TOR), and Sortis (SOR) were purchased from a local pharmacy. The composition of the two pharmaceutical products is in the case of: (1) TOR tablets—20 mg ATC, microcrystalline cellulose nucleus, MgO, lactose monohydrate, croscarmellose sodium, low substitution hydroxypropyl cellulose, anhydrous colloidal SiO_2_, magnesium stearate; film—hypromellose, macrogol 6000, TiO_2_ and talc; and (2) SOR tablets—20 mg ATC, CaCO_3_, microcrystalline cellulose, lactose monohydrate, croscarmellose sodium, polysorbate 80, hydroxypropyl cellulose, and magnesium stearate.

Using aqueous solutions of Na_2_HPO_4_ and NaH_2_PO_4,_ PBs with pH equal to 7 and 8 were prepared. In order to highlight the photodegradation of ATC, aqueous solutions with the concentrations of 3.58 mM (2 mg/ml), 1.79 mM (1 mg/ml) and ~ 0.9 mM (0.5 mg/ml) were prepared. In the case of the two drugs, the TOR and SOR tablets were ground in order to be dispersed in PB with pH 7 and 8, respectively, ultrasonicated for 30 min and finally filtered, when clear solutions were obtained.

The photoluminescence (PL) and photoluminescence excitation (PLE) spectra of ATC, TOR and SOR in solid state and as solutions were recorded, in right-angle geometry, with a Fluorolog-3 spectrometer, model FL3-22, from Horiba Jobin Yvon, having as excitation source a Xe lamp with the power of 450 W. The excitation and emission wavelengths used for the recording of the PL and PLE spectra, were equal to 275 and 420 nm, respectively.

Raman spectra of ATC before and after their interaction with NaOH were recorded with a FT Raman spectrophotometer, RFS100S model, from Bruker, endowed with a YAG:Nd laser.

IR spectra of ATC before and after its interaction with NaOH were recorded, in the attenuated total reflection geometry, with a FTIR spectrophotometer, Vertex 70 model, from Bruker.

The XPS spectra of ATC before and after the exposure to UV light, time of 216 min, were recorded with a SPECS spectrometer having an electron energy analyzer of the type Phoibos 150, working in a mode of Fixed Analyzer Transmission under an ultra-high vacuum of cca. 10^–7^ Pa. A monochromatic X-ray source of the type XR-50M with Al anode (Al Kα 1486.74 eV) was used. The acquisition of XPS spectra was carried out over 20 eV ranges at a pass energy of 10 eV, the energy resolution being of 0.07 eV.

In the case of the studies of Raman scattering, FTIR spectroscopy and XPS, the ATC photodegradation experiments were carried out with a mercury-vapor lamp, its power being of 350 W, the Hg spectral line of high intensity being at 253 nm.

## Supplementary Information

Supplementary Figures.
