# Supplementary material for: Spectroscopic studies on photodegradation of atorvastatin calcium
Source: Sci Rep. 2021 Jul 28;11:15338. doi: 10.1038/s41598-021-94693-5 (PMC8319406; doi:10.1038/s41598-021-94693-5)
Supplement: Supplementary file 1 — Supplementary Figures. [file 41598_2021_94693_MOESM1_ESM.docx]

**Supplementary Information**

**Spectroscopic studies on photodegradation of atorvastatin calcium**

*Madalina Oprica^1^, Miruna Iota^1^, Monica Daescu^1^, Szilard N. Fejer^2^, Catalin Negrila^3^, Mihaela Baibarac^1*^*

^1^National Institute of Materials Physics, Laboratory of Optical Processes in Nanostructured Materials, Atomistilor street 405A, Magurele P.O. Box MG-7, RO77125, Romania

^2^Pro-Vitam Ltd., Muncitorilor street 16, Sfantu Gheorghe, Romania

^3^National Institute of Materials Physics, Nanoscale Condensed Matter Laboratory, Atomistilor street 405A, Magurele P.O. Box MG-7, RO77125, Romania

***** E-mail address: [barac@infim.ro](mailto:barac@infim.ro)

| ****  **(a_1_)** | ****  **(a_2_)** |
| --- | --- |
| ****  **(b_1_)** | ****  **(b_2_)** |

**Figure 1S**. PLE (1) and PL (2) spectra of TOR (**a_1_, a_2_**) and SOR (**b_1_, b_2_**) before and after exposure to UV light for 216 min.

|  (**a_1_**) |  (**a_2_**) |
| --- | --- |
|  (**b_1_**) |   (**b_2_**) |

**Figure 2S.** PLE and PL spectra of the aqueous solution of ATC having the concentration 1 mg/ml (**a_1_**, **a_2_**) and 2 mg/ml (**b_1_, b_2_**) and their variations under UV light for 216 min.

(**1**)

(**2**)

**Figure 3S**. The photochemical reaction of: ATC in the presence of water vapors from air (**1**) and atorvastatin with oxygen from air (**2**).

| ****  **a_1_** |   **a_2_** |
| --- | --- |
|   **b_1_** |   **b_2_** |
|   **c1** |   **c_2_** |
|   **d_1_** |   **d_2_** |
|   **e1** |   **e_2_** |

**Figure 4S**. The XPS C1s (**a**), O1s (**b**), N1s (**c**), Ca2p (**d**) and F1s (**e**) spectra of ATC before (**a_1_**, **b_1_**, **c_1_**, **d_1_**, **e_1_**) and after the exposure to UV light (**a_2_, b_2_, c_2_, d_2_, e_2_**).

|  (**a**) |  (**b**) |
| --- | --- |

**Figure 5S.** PLE (**a**) and PL (**b**) spectra of the ATC 2 mg/ml interacted with the solution of NaOH 1.5M and their evolution under UV light, time of 216 min.

(**1**)

(**2**)

**Figure 6S**. The photoreaction of ATC with NaOH in the molar ratio 1:2 (**1**) and 1:4 (**2**).
